# Age‐Related Plasticity Integration in Male Cicadas *Tettigetta isshikii*: Are Adult Cicadas Capital Breeders?

**DOI:** 10.1002/ece3.70569

**Published:** 2024-11-11

**Authors:** Jiman Heo, Chang S. Han

**Affiliations:** ^1^ Department of Biology Kyung Hee University Seoul Korea; ^2^ Korea Institute of Ornithology Kyung Hee University Seoul Korea

**Keywords:** ageing, capital breeding, cicada, plasticity, *Tettigetta isshikii*, within‐individual correlation, within‐individual variation

## Abstract

Labile traits, such as behavioural, physiological traits or body mass, vary within an individual either over time or across environments. Such changes within an individual can be linked across traits, forming within‐individual trait correlations. These correlations are particularly expected when ageing causes changes in the expression of multiple traits. Moreover, the direction of these correlations depends on mechanisms that explain age‐related changes in each trait, such as physiological deterioration or changes in future fitness expectations. Therefore, assessing within‐individual trait correlations offers insights into trait‐specific ageing patterns, their integration and age‐related reproductive strategies. Here, we tracked individual male cicadas (*Tettigetta isshikii*) in their natural habitat, repeatedly assessing their plant use (narrow‐leafed vs. large‐leafed plants), calling activity and body mass. The results revealed that male cicadas lost mass, increased calling activity and a preference for narrow‐leafed plants as they aged. This integration of age‐related plasticity led to negative within‐individual correlations between body mass and behaviours. Considering that adult cicadas consume nutritionally poor xylem sap, the negative within‐individual correlations between body mass and risk‐taking behaviour suggest that *T. isshikii* males follow a capital breeding strategy rather than an income breeding strategy. As adult cicadas may use up the energy stored during the nymph stage, an age‐related increase in energetically demanding calling activity could cause an age‐related decrease in body mass. The terminal investment hypothesis could also explain the age‐related increase in calling activity and the preference for narrow‐leafed plants. Therefore, we emphasise the importance of individual‐level tracking studies in the wild to achieve a comprehensive understanding of the life‐history strategies and behavioural ecology of a study animal.

## Graphical abstract

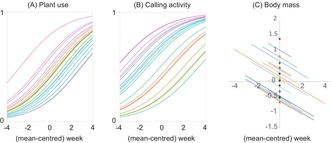

## 1 Introduction

**FIGURE 1:**
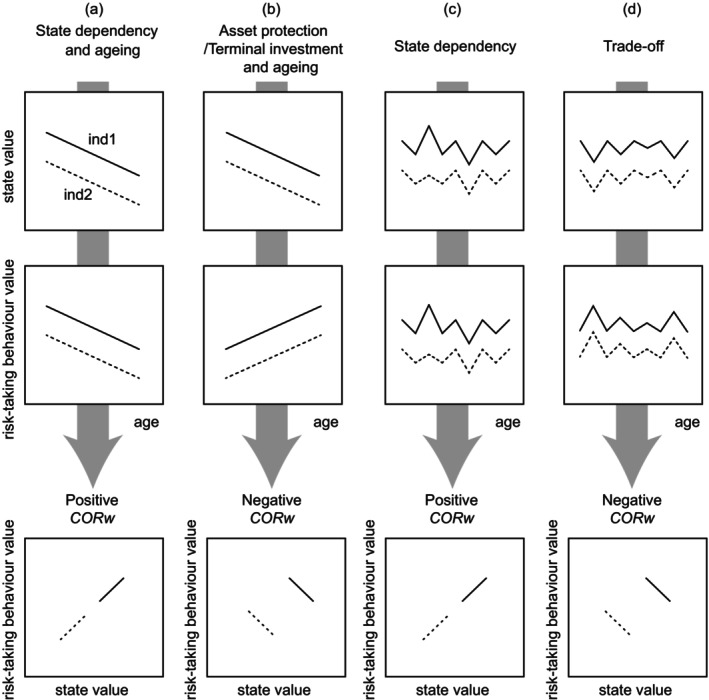
Hypothetical scenarios shaping within‐individual correlations (*CORw*) between state and risk‐taking behaviour. Temporal changes in traits, specifically related to ageing, strongly shape within‐individual trait correlations (a, b). (a) A positive within‐individual trait correlation is expected when both individual states and behaviours decrease with age due to physiological deterioration. (b) A negative within‐individual trait correlation is expected when individuals are more likely to engage in risk‐taking behaviour with decreasing future fitness expectations with age. However, within‐individual trait correlations can occur even when traits do not change with age but fluctuate based on (c) state dependence or (d) trade‐offs.

Within an individual, the expression of labile traits (e.g., behavioural, physiological or morphological traits such as body mass) can exhibit variation with individuals adjusting their phenotypes over time or in response to environmental changes. Moreover, within‐individual changes in multiple traits can also be associated across traits (Dingemanse, Dochtermann, and Nakagawa 2012; Dingemanse and Dochtermann 2013). If the expression of multiple traits is based on a shared underlying mechanism or if these traits affect each other via a feedback loop (Sih et al. 2015), we would expect to find an association between within‐individual changes in multiple traits. For instance, if exercise elevates the plasma level of corticosterone in mice (Girard et al. 2002), the daily pattern of exercise is expected to align with that of corticosterone levels. In addition, as a high metabolic rate is needed to express energetically demanding behaviour (such as risk‐taking behaviour), within‐individual changes in resting metabolic rate are expected to parallel those in risk‐taking behaviour (e.g., Cornwell, McCarthy, and Biro 2020). The correlation between changes in multiple traits within an individual is termed a ‘within‐individual trait correlation’ (Dingemanse and Dochtermann 2013). Multiple traits may change (or fluctuate) in the same direction within an individual, causing a positive within‐individual trait correlation (scenarios 1 and 3 in Figure 1). Conversely, traits may change (or fluctuate) in opposite directions within an individual, resulting in a negative within‐individual trait correlation (scenarios 2 and 4 in Figure 1).

Within‐individual trait correlations are strongly expected when trait expression is dependent on age (scenarios 1 and 2 in Figure 1). Notably, since individual differences in behaviour can stem from variations in state variables (Biro and Stamps 2008; Dingemanse and Wolf 2010; Wolf and Weissing 2010; Sih et al. 2015), age‐related decreases in labile state variables (e.g., body mass, metabolic rate and hormone levels) are expected to correlate with decreases in behaviours, leading to positive within‐individual correlations between state variables and behaviours (scenario 1 in Figure 1). In this scenario, physiological deterioration with ageing may decrease state trait levels, subsequently reducing the expression of state‐dependent behavioural traits and resulting in a positive within‐individual correlation between state and behaviour. For example, in the male field cricket *Gryllus campestris*, both body mass and exploration decrease with age, leading to a positive within‐individual correlation between body mass and exploration (Santostefano et al. 2016). In addition to ageing, there may be a positive feedback loop between body mass and exploration in the field cricket (e.g., reduced body mass decreases exploration, and vice versa), further contributing to a positive within‐individual correlation between body mass and exploration.

In addition, within‐individual trait correlation can be an outcome of age‐related decreases in future fitness expectations (i.e., residual reproductive value, Williams 1966). As older individuals have low residual reproductive value (i.e., reduced expectations of future reproduction), they are more likely to exhibit risk‐taking behaviour (e.g., Dammhahn 2012; Fisher, David, et al. 2015; Fisher, James, et al. 2015; Ory, van Son, and Thiel 2015; Moschilla, Tomkins, and Simmons 2018) and invest more into the current reproduction (terminal investment hypothesis, Williams 1966; Clutton‐Brock 1984). In contrast, younger individuals with higher residual reproductive value would not tend to take risks because their reproductive costs from injury or death are greater than the ones of older individuals (asset protection hypothesis, Clark 1994; Luttbeg 2017). This could potentially result in strongly positive within‐individual correlations among risk‐taking behaviours. Moreover, if age‐related decreases in state variables such as body mass reflect changes in future fitness expectations (e.g., body mass determines dominance), a decrease in body mass with age would be integrated with the age‐related increase in multiple risk‐taking behaviours, resulting in a negative within‐individual correlation between body mass and risk‐taking behaviour (scenario 2 in Figure 1).

Overall, the direction of within‐individual trait correlations between state and behaviour can vary depending on the trait‐specific mechanisms leading to age‐related increases/decreases or fluctuations (Figure 1). Recent meta‐analyses have indicated a weak relationship between state and behaviour at both the within‐individual and among‐individual levels (Niemelä and Dingemanse 2018); however, this finding might be attributed to a paucity of studies conducting frequent repeated measurements of labile traits with longer intervals between the repeated measurements. Therefore, it is imperative to assess within‐individual trait correlations to identify the underlying trait‐specific or common mechanisms shaping age‐related plasticity. However, within‐individual correlations have attracted considerably less attention from behavioural ecologists (Dochtermann 2023).

**FIGURE 2:**
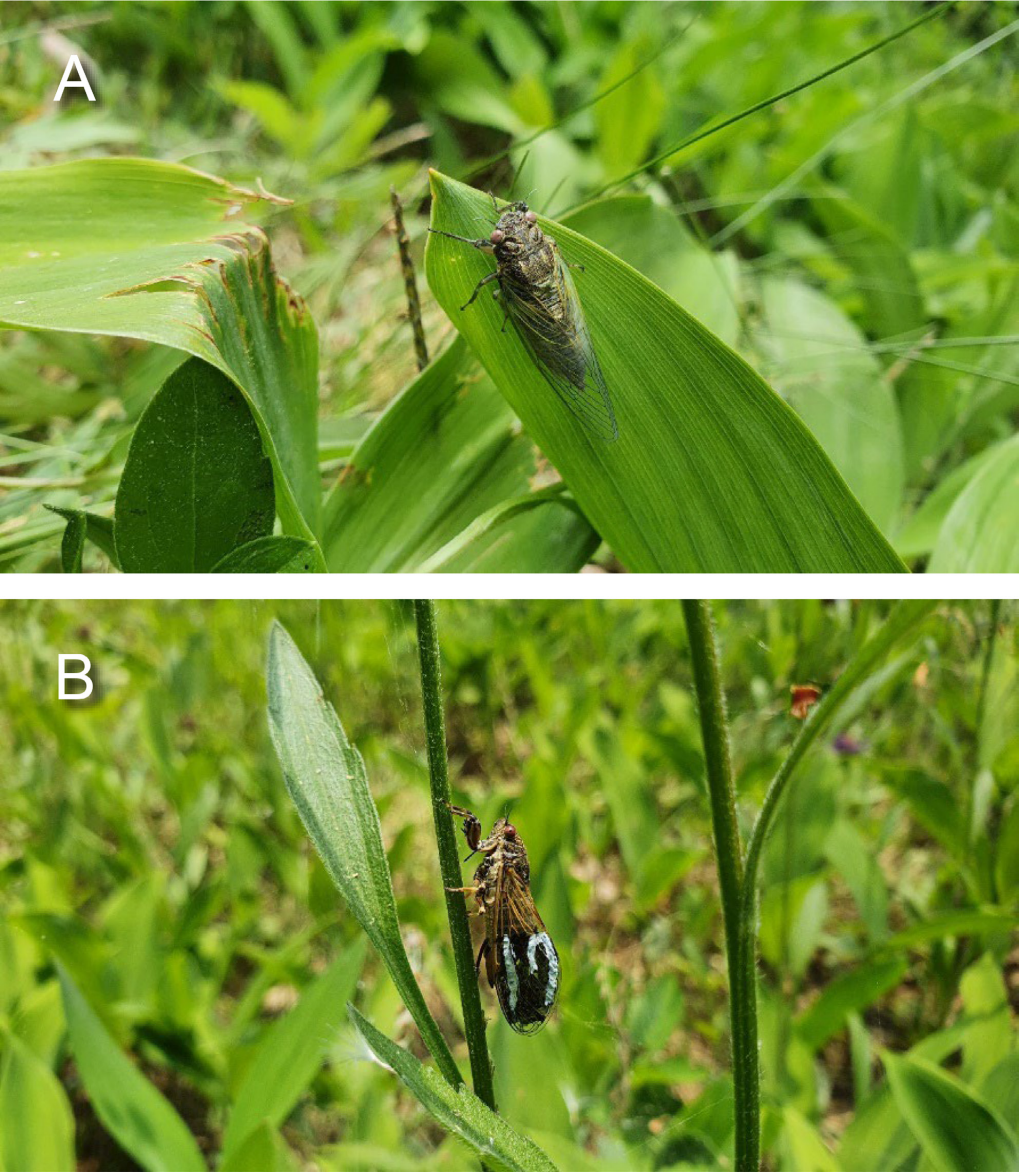
(A) A *Tettigetta isshikii* individual resting on a *Convallaria majalis* leaf. (B) *T. isshikii* individuals were marked with enamel paint on their wings. (Photo credit: Jiman Heo).

**FIGURE 3:**
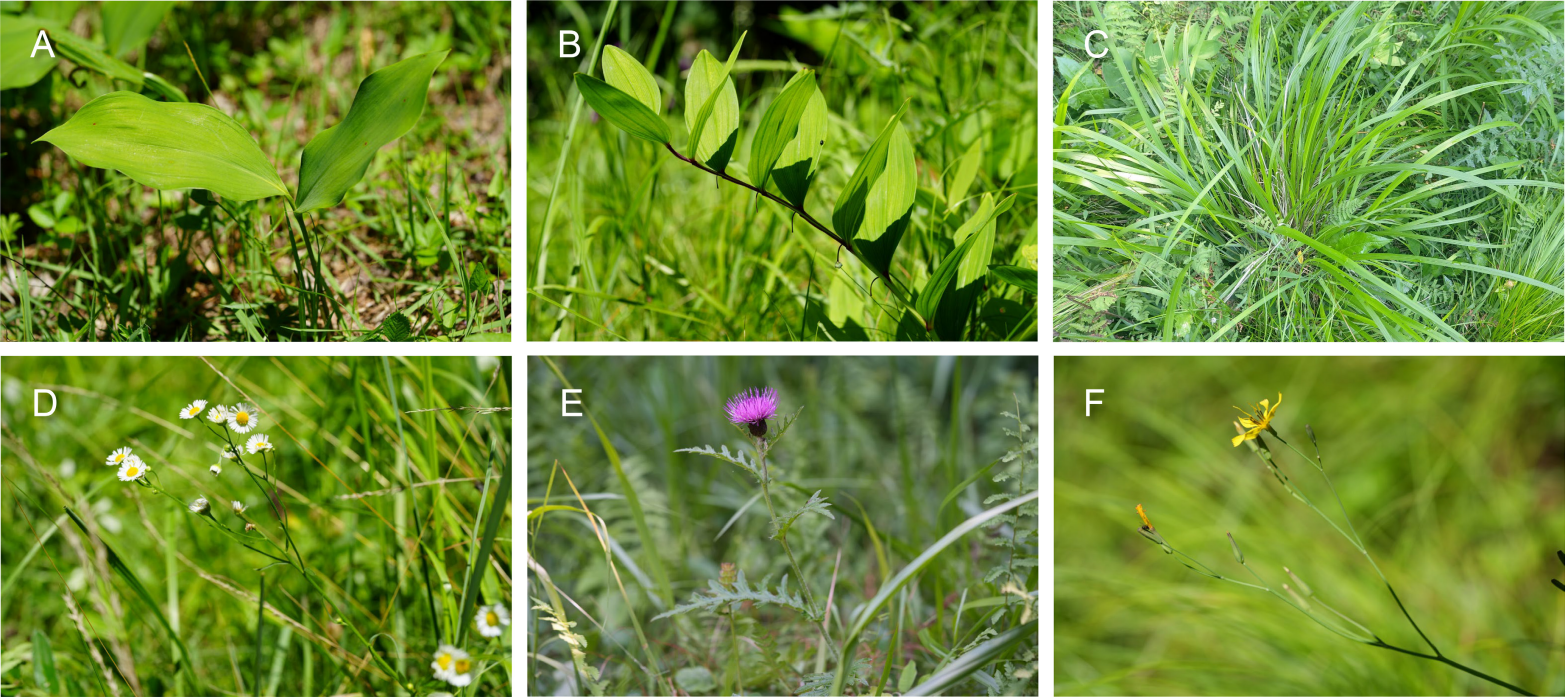
The plant species on which cicadas *Tettigetta isshikii* are found in their natural habitat. Large‐leafed plants: (A) *Convallaria majalis* and (B) *Polygonatum odoratum* var. *pluriflorum*; Narrow‐leafed plants: (C) *Miscanthus sinensis* , (D) *Erigeron annuus* , (E) *Cirsium japonicum* and (F) *Ixeridium dentatum* (Photo credit: Jiman Heo, Myeong Dong Cho).

In this study, we tracked individual male cicadas *Tettigetta isshikii* (Figure 2) in their natural habitat and repeatedly assessed their plant use, calling activity and body mass. We investigated age‐related changes in these traits, partitioned trait variance into the among‐ and within‐individual levels and estimated among‐ and within‐individual correlations among these traits. In this study, we predicted that two behaviours, plant use and calling activity, would reflect the boldness of each male. Cicadas actively use plants as shelters, adjusting their location within the plant to hide themselves (Steward, Smith, and Stephen 1988). Different plant species have different structural characteristics, such as leaf size and density, suggesting the shelter they provide for cicadas may differ by species. Cicadas might prefer plants with larger, denser leaves to protect them from aerial predators (e.g., robber flies). *T. isshikii* also feeds on herbaceous plant sap and lays its eggs in plant stems, potentially using the plants as shelters by hiding underneath the leaves (Jiman Heo, personal observation). In our study area, plants such as *Erigeron annuus* (Daisy fleabane), *Thalictrum aquilegiifolium* (Meadow rue) or *Miscanthus sinensis* (Chinese silver grass) have narrow or small leaves, whereas only two plants, *Convallaria majalis* (Lily of the valley) and *Polygonatum odoratum* (Solomon's seal), have large leaves and occurs in discrete clusters (Figure 3). As a result, only *C. majalis* and *P. odoratum* may effectively function as shelters for *T. isshikii*. Thus, we predicted that the increased use of narrow‐leafed plants might indicate greater boldness in the face of potential predators. In addition, male *T. isshikii* produces calls to attract females, and females respond with wing‐flicking sounds (Jiman Heo, personal observation). However, calling songs of cicadas increases the risk of predation by predators, such as the robber fly (Hou et al. 2017), suggesting that calling can also be considered a risk‐taking behaviour of male *T. isshikii*.

We predicted that trait‐specific mechanisms shaping age‐related plasticity would determine within‐individual trait correlations. If physiological deterioration leads to both reductions in calling activity and boldness with age as well as weight loss in male cicadas after eclosion and maturation, positive within‐individual correlations between body mass and risk‐taking behaviours are expected (scenario 1 in Figure 1). In contrast, according to the terminal investment hypothesis, male cicadas might increase the expression of risk‐taking behaviours with age, even as body mass decreases (scenario 2 in Figure 1). This scenario could lead to negative within‐individual correlations between body mass and risk‐taking behaviours (scenario 2 in Figure 1). Consequently, we predicted that the integration of age‐related plasticity in multiple traits of male cicadas would hinge on the trait‐specific mechanisms governing age‐related changes.

## 2 Materials and Methods

### 2.1 Study Species

*Tettigetta isshikii* (Hemiptera: Cicadettinae) is a small cicada species distributed across East Asia, including Korea, Russia (Sakhalin, Ussuri and Siberia) and China (Lee 2008) (Figure 2A). In Korea, *T. isshikii* is widespread on the mainland but not on small islands, except for Jeju Island (Lee 2008). The species is found at various altitudes, ranging from 30 m to as high as 1000 m (Lee, Oh, and Park 2004; Jiman Heo, personal observation). Since *T. isshikii* adults feed on xylem sap from herbaceous plants and lay eggs in the stems of herbaceous plants, they are rarely found in trees (Jiman Heo, personal observation). During the day, adult male *T. isshikii* produce long calls to attract females, but they remain silent at night (Lee, Oh, and Park 2004; Jiman Heo, personal observation). Based on our observation, assuming that the cicadas were alive on the day on which they were found, the average longevity of adult male *T. isshikii* was 2.47 days (SE = 0.32, *N* = 42), with a maximum observed lifespan of 9 days. This study is the first to measure the lifespan of *T. isshikii*, although the estimates should be interpreted with caution because not recapturing an individual does not necessarily mean it has died.

### 2.2 Study Site

The field observations were carried out in a grassy field located in PyeongChang‐gun, Gangwon‐do, Korea (37°34′53.9″ N 128°24′58.6″ E, altitude: 590 m). The site was a rectangular grassy field (area: 30 × 8 m = 240 m^2^) containing diverse herbaceous plants. The field was enclosed by oak and pine trees, and cicadas were exclusively observed within this area. Throughout the observations, we measured the temperature and humidity with a thermometer and humidity meter (Testo 174H Mini data logger, Germany). Notably, the temperature and humidity variations during the three observation periods (detailed below) within a day corresponded closely to the observation time (i.e., the temperature always increased, but the humidity decreased from the morning to the mid‐afternoon observation period) (Figure S1). As a result, temperature and humidity were excluded from the subsequent analysis.

### 2.3 Field Observations and Morphological Measurements

Observations were conducted three times per day (from 09:00 to 10:00, 12:00 to 13:00 and 15:00 to 16:00) between 1st June and 13th June, 2022, excluding 6th June due to rain. Consequently, the maximum count of daily observations for each individual was three or fewer. During the observation periods, as the observer walked through the habitat, whenever we found individuals, we captured them using a net, weighed them using a scale (Pocket Scale MH‐100) to the nearest 0.01 g, and subsequently released them at their original location. Previously unmarked (newly found) individuals were individually marked on their left wing with a paint marker (PC–3 M, POSCA) to allow the identification of these individuals (Figure 2B).

**FIGURE 4:**
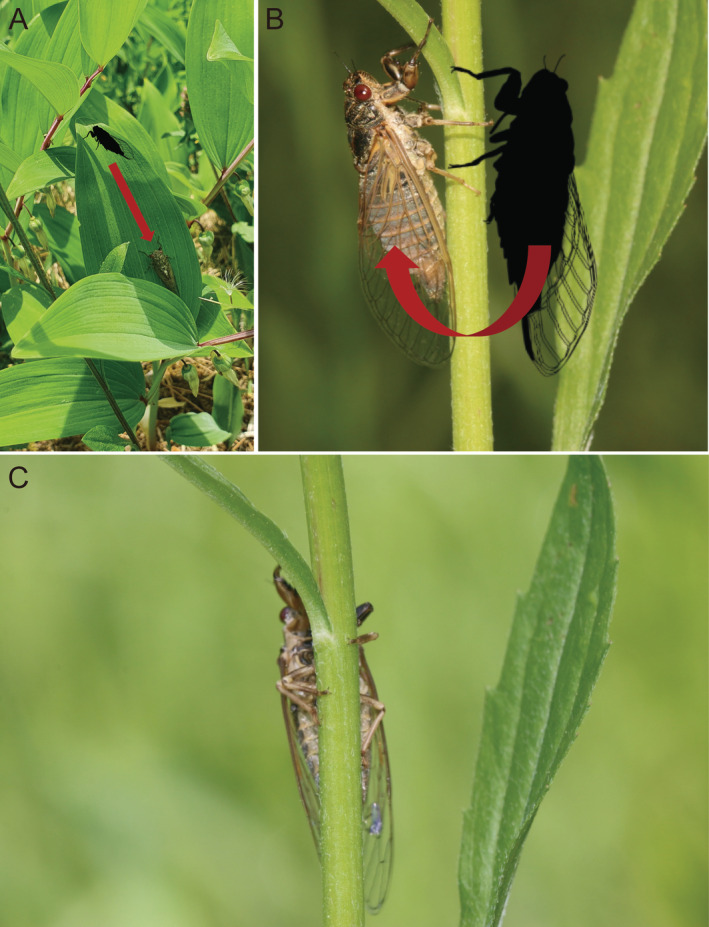
*Tettigetta isshikii* uses plant structures to hide from predators. (A) When *T. isshikii* on the tip of a leaf from *Polygonatum odoratum* var. *pluriflorum* (large‐leafed plant) felt threatened, it moved towards the base or underside of the leaf to hide. (B, C) In contrast, when *T. isshikii* on the stem of *Erigeron annuus* (narrow‐leafed plant) felt threatened, it moved behind the stem.

When we found cicadas, we also recorded the plant species on which the individuals were located. In the study area, cicadas were found on multiple herbaceous plants such as *Convallaria majalis* (Lily of the Valley), *Polygonatum odoratum* var. *pluriflorum* (Solomon's seal), *Miscanthus sinensis* (Chinese silver grass), *Erigeron annuus* (Daisy fleabane), *Cirsium japonicum* (Japanese thistle) and *Thalictrum aquilegiifolium* var. *sibiricum* (Meadow rue) (Figure 3). Among the plants, only *C. majalis* and *P. odoratum* have large leaves, and we predict that these two species can effectively function as shelters for *T. isshikii*. Based on our observation, when *T. isshikii* felt threatened, it either flew away or hid beneath a leaf or behind a stem. Specifically, when *T. isshikii* on the tip of a large‐leafed plant felt threatened, it moved towards the base or underside of the leaf to hide (Figure 4A). In contrast, when *T. isshikii* on the stem or leaf of small‐leafed or narrow‐leafed plants felt threatened, it moved behind the stem or beneath the leaf (Figure 4B). This suggests that leaf size might influence the probability of being attacked by predators such as robber flies. Larger leaves could provide safer shelters for *T. isshikii*. In addition to leaf size, the vertical position of cicadas on a plant (e.g., near the ground vs. near the tip) can indicate a risk‐taking tendency. Cicadas near the tip may be bolder, as they are more exposed and vulnerable to predators. However, since cicadas are found on various plant species, their level of exposure may vary depending on the plant even at the same height from the ground. Thus, we simply focused on whether the cicadas were found in large‐leafed ( *C. majalis* and *P. odoratum*) or small‐leafed and narrow‐leafed plants (other plant species).

During the observation, we also recorded whether the males produced calls. *T. isshikii* males consistently produce calls regardless of the presence of a quietly walking observer (Jiman Heo, personal observation). Males were assigned a score of 1 when they were engaged in calling and a score of 0 when they were silent.

Although we marked 41 males and 23 females in the field, we failed to sample a sufficient number of female individuals and lacked sufficient observations to conduct variance partitioning analyses. Thus, in this study, we focused on analyses of data collected from males. We collected 103 behavioural and morphological samples from males, and repeated measurements were performed 2.45 times on average per individual.

### 2.4 Statistical Analyses

Our first set of analyses using univariate mixed‐effects models examined individual differences in trait expression and how trait expression changed with age or time within a day. Our second set of analyses used bivariate mixed‐effect models to estimate within‐sex among‐ and within‐individual correlations between traits. We fitted all the univariate and bivariate models within a Bayesian framework using the MCMCglmm package (Hadfield 2010) in R (version 3.2.0). To minimise autocorrelation among samples, 1,300,000 Markov Chain Monte Carlo (MCMC) iterations were performed, which were sampled at 1000‐iteration intervals after an initial burn‐in period of 300,000 iterations, using Gamma priors. This resulted in a total of 1000 samples from the posterior distribution. Convergence was attained by visual inspection of output plots and by ensuring that autocorrelation between consecutive samples did not exceed 0.1 (Hadfield 2010).

#### 2.4.1 Univariate Models

The univariate models fitted individual identity as a random effect and (within‐individual mean‐centred) observation day and (mean‐centred) time of day as covariates. We used the following error structure for the response variable: body mass was modelled with Gaussian errors, and plant use (found on narrow‐leafed plants [1] or large‐leafed plants [0]) and calling activity (exhibiting calling [1] or not [0]) were modelled with binomial errors. Despite including a quadratic term of observation day (i.e., age) as an additional covariate in the univariate models, we did not observe quadratic ageing patterns in any of the traits (results not included). Consequently, the univariate models did not include a quadratic term for the observation day. In addition to the random intercept model, we used a random slope model to estimate among‐individual variation in age‐related plasticity. We found no variation in the age‐related slopes (results not included), which may be due to the limited sample size in our study (a small number of individuals with few repeated measures).

#### 2.4.2 Bivariate Models

We constructed sets of bivariate models, where we fitted two traits from plant use, calling activity and body mass as the response variables. These models included individual identity as a random effect and had no fixed effects. This allowed within‐individual trait variances to reflect age‐related changes, enabling us to test how within‐individual age‐related changes were associated across different traits. In the bivariate models, we constrained the among‐individual covariance between body mass and behavioural traits to zero because the among‐individual variances in behaviours were not significantly positive (see Section 3). The within‐individual variances in plant use and calling activity were fixed to one because they were not estimable with binary data. Thus we were not able to calculate a within‐individual covariance between calling activity and plant use.

## 3 Results

**FIGURE 5:**
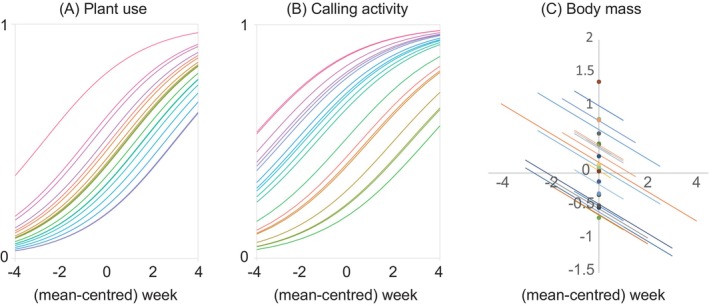
Temporal changes in the expression of (A) plant use, (B) calling activity and (c) body mass of male cicadas *Tettigetta isshikii*. In the plant use panel (A), 1 indicates that the cicada was found on narrow‐leafed plants, whereas 0 indicates that the cicada was found on large‐leafed plants. In the calling activity panel (B), 1 indicates that the cicada was calling, whereas 0 indicates that the cicada was not calling. Different coloured lines indicate different individuals.

Body mass varies among individual cicadas (Table 1). However, the use of narrow‐leafed plants and the expression of calling activity did not tend to vary among individual cicadas (Table 1, Figure S2). Behaviours were not a function of the time of day (time of day effect, Table 1). However, with repeated observations, cicadas lost body mass, were found on narrow‐leafed plant species and increased calling activity (age effect, Table 1, Figure 5). These age‐related plasticities were reflected in within‐individual trait covariations: the 95% CIs of within‐individual covariations between body mass and behaviours were smaller than zero (Table 2).

**TABLE 1:** Age‐related plasticity and individual differences in the plant use, calling activity and body mass of male cicadas. Point estimates are provided with 95% credible intervals (95% CIs) in parentheses.

	Plant use^a^	Calling activity	Body mass
Fixed effects	β (95% CI)	β (95% CI)	β (95% CI)
Intercept	−0.65 (−1.38, 0.08)	0.05 (−0.88, 0.91)	0.12 (−0.16, 0.40)
Time of day^b^	0.66 (−0.14, 1.44)	0.11 (−0.77. 0.90)	
Age^c^	0.49 (0.06, 1.00)	0.45 (0.06, 0.83)	−0.22 (−0.34, −0.11)
Random effects	σ^2^ (95% CI)	σ^2^ (95% CI)	σ^2^ (95% CI)
ID	1.52 (0.13, 4.04)	3.62 (0.12, 9.85)	0.54 (0.21, 0.98)
Residual	1.00 (−)	1.00 (−)	0.47 (0.27, 0.71)

^a^ Tendency to be found in narrow‐leafed plants.

^b^ Within‐group centred time of day (−1 = 09:00–10:00, 0 = 12:00–13:00 and 1 = 15:00–16:00).

^c^ Within‐individual mean‐centred testing day.

**TABLE 2:** Within‐individual trait variances and covariances.

	Within‐individual variance (95% credible interval)
Body mass	0.68 (0.41, 1.00)
Plant use^a^	1.00 (−)
Calling activity	1.00 (−)

	Within‐individual covariance (95% credible interval)
Body mass—Calling activity	−0.42 (−0.74, −0.04)
Body mass—Plant use	−0.40 (−0.78, −0.07)
Plant use—Calling activity	NA

^a^ Tendency to be found in narrow‐leafed plants.

## 4 Discussion

We demonstrated that male cicadas exhibited a decrease in body mass, an increase in calling activity and moved to open habitats (narrow‐leafed plants) with age. Such integration of age‐related plasticity resulted in negative within‐individual covariations between states (body mass) and risk‐taking behaviours. This pattern may be attributed to the combined effects of terminal investment and energetically demanding mate calling. The age‐related decrease in body mass might be due to an age‐related increase in calling efforts, and the age‐related increase in risk‐taking behaviours might be due to decreased future fitness expectations with age. Moreover, the integration of age‐related plasticity in *T. isshikii* implies that *T. isshikii* uses a capital breeding reproductive strategy.

Considering the age‐related increase in calling activity, physiological deterioration may not occur in wild cicadas, although this remains uncertain. This age‐related increase in calling activity might be due to the immaturity of younger males, who are not yet capable of producing calls. However, a quadratic ageing pattern was not evident in male calling activity, as the increased calling activity did not decrease with age. These findings suggest that the changes in calling activity are not due to physiological deterioration. Nevertheless, the possibility of physiological deterioration cannot be entirely excluded. Despite thorough searches of the habitat, it is possible that noncalling older males were missed, potentially biasing the analysis of age‐related changes in calling activity. For instance, a nonlinear ageing pattern in calling behaviour might exist, but our study might have failed to detect it if nonsinging old males were overlooked during our observations.

The age‐related increase in risk‐taking behaviour among male *T. isshikii* can be explained by the terminal investment hypothesis (Williams 1966; Clutton‐Brock 1984) and asset protection principle (Clark 1994). Our analysis of plant use revealed that older males were more often found on narrow‐leafed or small‐leafed plant species. Although our data do not confirm that plants with different leaf sizes perform differently as shelters for *T. isshikii*, young adult males may safely hide in large‐leafed plants. As male cicadas age, they may prefer to stay on narrow‐leafed plants to increase their body temperature and calling activity, actively advertising their locations to females. Following the asset protection principle, male cicadas stay on large‐leafed plants to maximise protection with no courtship activity during early adulthood but move to narrow‐leafed plants to actively seek mates during late adulthood. These behavioural strategies likely maximise the reproductive success of male cicadas during their short reproductive periods.

The age‐related decrease in body mass among cicadas might be attributed to the stress from repeated net captures. Although we minimised disturbance by quickly confirming their identity before release, repeated netting could still potentially contribute to a decline in body mass over time. Moreover, if the stress from repeated captures and the principle of asset protection are both active, this stress could reduce body mass and subsequently lead to increased risk‐taking behaviours. Therefore, we cannot completely rule out the possibility that our collection methods may influence the ageing process of cicadas, potentially affecting the within‐individual correlations between body mass and risk‐taking behaviours.

Instead of the role of stress in shaping within‐individual correlations between body mass and risk‐taking behaviours, the age‐related decrease in body mass and increase in risk‐taking behaviour in male cicadas may imply resource allocation strategies for reproduction. There are two extreme resource allocation strategies according to the timing of reproduction: capital breeding and income breeding (Drent and Daan 1980; Jönsson and Jonsson 1997). Capital breeders accumulate resources before the reproductive period and utilise these resources during reproduction, whereas income breeders depend on current resource acquisition during reproduction (Jönsson and Jonsson 1997). For insect capital breeders, nymphs (or larvae) grow their bodies, accumulate resources over a long period and then spend their stored energy in a short reproductive period after they eclose to adulthood (Boggs 1992). For example, flightless females of the common glowworm *Lampyris noctiluca* emit light to attract males using energy from resources that were accumulated during the larval stage (Hopkins et al. 2015). Larval nutritional conditions influence adult phenotypes and life history (reviewed in Scriber and Slansky 1981; Awmack and Leather 2002; Han and Dingemanse 2015; Koyama and Mirth 2018), although many insects consume macronutrients during the reproductive period and use resources acquired during the adult stage directly for reproduction (i.e., income breeders). Some insect species are recognised as capital breeders (Tammaru and Haukioja 1996; Boggs 1997; Kemp and Alcock 2003; Casas et al. 2005; Johnson 2006; Bauerfeind and Fischer 2008; Rhainds, Leather, and Sadof 2008; Pöykkö 2009; Davis et al. 2016). Considering the continuum from pure capital breeding to pure income breeding, many insect species are inclined towards being income breeders. However, cicadas have not been extensively studied in this context. The findings of our study suggest that *T. isshikii* may be capital breeders. First, the age‐related decrease in the body mass of adult *T. isshikii* supports this notion. Capital breeders use resources stored during the nymph stage, leading to a sharp decrease in body mass with age during the reproductive period, whereas income breeders can maintain or even increase their body mass with age (Molleman et al. 2022). For example, fruit‐feeding butterflies do not lose their body mass during reproductive periods, suggesting that they are income breeders (Molleman et al. 2022). In contrast, the age‐related changes in the body mass of adult *T. isshikii* showed the opposite pattern, indicating that these individuals may be capital breeders. However, adult *T. isshikii*, like other cicadas, are known to consume a substantial amount of xylem sap as adults (Cheung and Marshall 1973). Despite this intake, the sharp decrease in the body mass of male *T. isshikii* occurs because the plant sap consumed by cicadas is nutritionally poor. Both nymph and adult cicadas feed on xylem (Brown and Chippendale 1973; Cheung and Marshall 1973; White and Strehl 1978), which contains mainly water (99%) with very low concentrations of nutrients such as carbohydrates, amino acids and proteins (Satoh et al. 1992; Dafoe and Constabel 2009). Water intake may be the primary aim when adult cicadas consume xylem sap because water loss across the cuticle is high in cicadas (Hepler et al. 2023). Instead, cicadas may collect nutrients during the nymphal stage, which is necessary for reproduction at the adult stage. Although nymphs also rely on nutritionally poor xylem sap for nutrient intake (Cheung and Marshall 1973; White and Strehl 1978), they can have enough time to accumulate reserves and grow (White and Lloyd 1975). Thus, adult cicadas seem to rely on resources that are stored during the larval stage for reproduction. In fact, female cicada fecundity is also known to depend on resource acquisition during the larval stage (Brown and Chippendale 1973).

Moreover, capital breeders are expected to show a negative within‐individual correlation between body mass and reproductive behaviour, which was found in our study of male *T. isshikii*. Unlike income breeders, capital breeders allocate their energy to reproduction using the limited resources accumulated during the nymphal stage, resulting in a short reproductive period and lifespan (Beck and Fiedler 2009; Holm et al. 2016). Consequently, capital breeders are expected to sharply increase their reproductive efforts after eclosion to adults. As the expression of energetically costly reproductive behaviour increases with age, the body mass of capital breeders decreases due to the depletion of stored resources, leading to a negative within‐individual age‐related correlation between body mass and reproductive behaviour. In contrast, income breeders continuously collect resources and use them directly during reproduction in the adult stage. Therefore, an age‐related increase in reproductive effort is not expected to cause a decrease in body mass, resulting in nonsignificant within‐individual age‐related correlations between body mass and reproductive behaviour. In our study, the short lifespan of *T. isshikii* adults (2.47 days) and the negative within‐individual correlation between body mass and calling activity in males strongly suggest that the capital breeding strategy is more prominent in *T. isshikii*. To further evaluate whether resource allocation strategies during cicada reproduction are more closely aligned with those of capital breeders along the income‐capital breeding continuum, future studies should investigate how food intake during the nymph and adult stages affects reproductive output.

Alternatively, if body mass influences agility in escaping predators in cicadas, this can also lead to within‐individual correlations between body mass and risk‐taking behaviours. In flying insect species, a greater body mass has been shown to reduce individual agility in escaping predators, increasing risk of predation (McLachlan, Ladle, and Crompton 2003; Roitberg, Mondor, and Tyerman 2003). Although the relationship between predation rate and body mass has not been assessed in cicada species, poor agility due to heavy body mass during early adulthood may make calling males more vulnerable to predators such as robber flies. Therefore, it might be advantageous for young males to reduce their calling activity and move cautiously. However, as body mass decreases with age, increased agility in escaping predators can reduce the predation risk of older males. This may enable older males to be more active in finding mates and producing calls. Thus, the relationship between body mass and the risk of predation may explain the within‐individual correlations between body mass and risk‐taking behaviours in our study.

While individual‐level behavioural studies with repeated measures experimental designs have been extensively conducted across various animal taxa in the wild (as reviewed in Hertel et al. 2020), such investigations are less common in wild insects (but see Fisher, James, et al. 2015; Fisher, David, et al. 2015; Niemelä, Lattenkamp, and Dingemanse 2015; Golab et al. 2021; Niemelä, Tiso, and Dingemanse 2021). This scarcity might arise from the challenges of distinguishing and tracking individual insects in their natural habitats. Despite a recent study demonstrating the presence of personality traits in cicadas under laboratory conditions (Roth et al. 2022), no studies have tracked individual cicadas in the wild, repeatedly measured their behaviour and assessed individual differences. Our field study addressed this gap, providing new insights into the behavioural ecology of cicadas, specifically shedding light on the reproductive and life history strategies of *T. isshikii* males. While we did not find significant individual differences in behaviours and their age‐related plasticity, this result may have occurred due to low statistical power because of the small sample size and limited number of repeated measurements per male. However, our study revealed the integration of age‐related plasticity in body mass and risk‐taking behaviours, suggesting a life‐history strategy of *T. isshikii* males. As adult cicadas rely on nutritionally poor xylem sap, the age‐related increase in energetically demanding behaviour (e.g., calling activity) may also cause an age‐related decrease in body mass, resulting in a negative within‐individual correlation between body mass and calling activity as well as a short lifespan. Taken together, these results suggest that *T. isshikii* males resemble capital breeders more than income breeders. Thus, we emphasise that studying individual behaviour in the wild is crucial for comprehensively understanding the behavioural ecology of the study animal and the evolutionary processes that shape their behaviours and life‐history strategies in the wild.

## Author Contributions

**Jiman Heo:** conceptualization (equal), data curation (equal), investigation (lead), writing – original draft (equal), writing – review and editing (equal). **Chang S. Han:** conceptualization (equal), data curation (equal), formal analysis (lead), funding acquisition (lead), methodology (lead), supervision (lead), writing – original draft (equal), writing – review and editing (equal).

## Conflicts of Interest

The authors declare no conflicts of interest.

## Supporting information


Figures S1–S2.

Data S1.

## Data Availability

All data is included as a Supporting Information.

## References

[ece370569-bib-0001] Awmack, C. S. , and S. R. Leather . 2002. “Host Plant Quality and Fecundity in Herbivorous Insects.” Annual Review of Entomology 47: 817–844. 10.1146/annurev.ento.47.091201.145300 11729092

[ece370569-bib-0002] Bauerfeind, S. S. , and K. Fischer . 2008. “Maternal Body Size as a Morphological Constraint on Egg Size and Fecundity in Butterflies.” Basic and Applied Ecology 9, no. 4: 443–451.

[ece370569-bib-0003] Beck, J. , and K. Fiedler . 2009. “Adult Life Spans of Butterflies (Lepidoptera: Papilionoidea + Hesperioidea): Broadscale Contingencies With Adult and Larval Traits in Multi‐Species Comparisons.” Biological Journal of the Linnean Society 96, no. 1: 166–184.

[ece370569-bib-0004] Biro, P. A. , and J. A. Stamps . 2008. “Are Animal Personality Traits Linked to Life‐History Productivity?” Trends in Ecology & Evolution 23, no. 7: 361–368. 18501468 10.1016/j.tree.2008.04.003

[ece370569-bib-0005] Boggs, C. L. 1992. “Resource Allocation: Exploring Connections Between Foraging and Life History.” Functional Ecology 6, no. 5: 508.

[ece370569-bib-0006] Boggs, C. L. 1997. “Dynamics of Reproductive Allocation From Juvenile and Adult Feeding: Radiotracer Studies.” Ecology 78, no. 1: 192–202.

[ece370569-bib-0007] Brown, J. J. , and G. M. Chippendale . 1973. “Nature and Fate of the Nutrient Reserves of the Periodical (17 Year) Cicada.” Journal of Insect Physiology 19, no. 3: 607–614.

[ece370569-bib-0008] Casas, J. , S. Pincebourde , N. Mandon , F. Vannier , R. Poujol , and D. Giron . 2005. “Lifetime Nutrient Dynamics Reveal Simultaneous Capital and Income Breeding in a Parasitoid.” Ecology 86, no. 3: 545–554.

[ece370569-bib-0009] Cheung, W. W. K. , and A. T. Marshall . 1973. “Water and Ion Regulation in Cicadas in Relation to Xylem Feeding.” Journal of Insect Physiology 19, no. 9: 1801–1816.

[ece370569-bib-0010] Clark, C. W. 1994. “Antipredator Behavior and the Asset‐Protection Principle.” Behavioral Ecology 5, no. 2: 159–170.

[ece370569-bib-0011] Clutton‐Brock, T. H. 1984. “Reproductive Effort and Terminal Investment in Iteroparous Animals.” American Naturalist 123, no. 2: 212–229.

[ece370569-bib-0012] Cornwell, T. O. , I. D. McCarthy , and P. A. Biro . 2020. “Integration of Physiology, Behaviour and Life History Traits: Personality and Pace of Life in a Marine Gastropod.” Animal Behaviour 163: 155–162.

[ece370569-bib-0013] Dafoe, N. J. , and C. P. Constabel . 2009. “Proteomic Analysis of Hybrid Poplar Xylem Sap.” Phytochemistry 70, no. 7: 856–863. 19467552 10.1016/j.phytochem.2009.04.016

[ece370569-bib-0014] Dammhahn, M. 2012. “Are Personality Differences in a Small Iteroparous Mammal Maintained by a Life‐History Trade‐Off?” Proceedings of the Royal Society B: Biological Sciences 279, no. 1738: 2645–2651. 10.1098/rspb.2012.0212 PMC3350711 22398164

[ece370569-bib-0015] Davis, R. B. , J. Javoiš , A. Kaasik , E. Õunap , and T. Tammaru . 2016. “An Ordination of Life Histories Using Morphological Proxies: Capital Versus Income Breeding in Insects.” Ecology 97, no. 8: 2112–2124. 27859210 10.1002/ecy.1435

[ece370569-bib-0016] Dingemanse, N. J. , and N. A. Dochtermann . 2013. “Quantifying Individual Variation in Behaviour: Mixed‐Effect Modelling Approaches.” Journal of Animal Ecology 82, no. 1: 39–54. 23171297 10.1111/1365-2656.12013

[ece370569-bib-0017] Dingemanse, N. J. , N. A. Dochtermann , and S. Nakagawa . 2012. “Defining Behavioural Syndromes and the Role of “Syndrome Deviation” in Understanding Their Evolution.” Behavioral Ecology and Sociobiology 66, no. 11: 1543–1548.

[ece370569-bib-0018] Dingemanse, N. J. , and M. Wolf . 2010. “Recent Models for Adaptive Personality Differences: A Review.” Philosophical Transactions of the Royal Society, B: Biological Sciences 365, no. 1560: 3947–3958. 10.1098/rstb.2010.0221 PMC2992752 21078647

[ece370569-bib-0019] Dochtermann, N. A. 2023. “The Role of Plasticity, Trade‐Offs, and Feedbacks in Shaping Behavioral Correlations.” Behavioral Ecology 34, no. 6: 913–918.

[ece370569-bib-0020] Drent, R. H. , and S. Daan . 1980. “The Prudent Parent: Energetic Adjustments in Avian Breeding 1.” Ardea 38–90: 225–252.

[ece370569-bib-0021] Fisher, D. N. , M. David , T. Tregenza , and R. Rodríguez‐Muñoz . 2015. “Dynamics of Among‐Individual Behavioral Variation Over Adult Lifespan in a Wild Insect.” Behavioral Ecology 26, no. 4: 975–985. 26167097 10.1093/beheco/arv048 PMC4495759

[ece370569-bib-0022] Fisher, D. N. , A. James , R. Rodríguez‐Muñoz , and T. Tregenza . 2015. “Behaviour in Captivity Predicts Some Aspects of Natural Behaviour, but Not Others, in a Wild Cricket Population.” Proceedings of the Royal Society B: Biological Sciences 282, no. 1809: 20150708. 10.1098/rspb.2015.0708 PMC4590455 26019161

[ece370569-bib-0023] Girard, I. , J. G. Swallow , P. A. Carter , P. Koteja , J. S. Rhodes , and T. Garland . 2002. “Maternal‐Care Behavior and Life‐History Traits in House Mice ( *Mus domesticus* ) Artificially Selected for High Voluntary Wheel‐Running Activity.” Behavioural Processes 57, no. 1: 37–50. 11864774 10.1016/s0376-6357(01)00206-6

[ece370569-bib-0024] Golab, M. J. , S. Sniegula , A. Antoł , and T. Brodin . 2021. “Adult Insect Personality in the Wild—Calopteryx Splendens as a Model for Field Studies.” Ecology and Evolution 11, no. 24: 18467–18476. 35003685 10.1002/ece3.8439 PMC8717306

[ece370569-bib-0025] Hadfield, J. D. 2010. “MCMC Methods for Multi‐Response Generalized Linear Mixed Models: The MCMCglmm R Package.” Journal of Statistical Software 33, no. 2: 1–22.20808728

[ece370569-bib-0026] Han, C. S. , and N. J. Dingemanse . 2015. “Effect of Diet on the Structure of Animal Personality.” Frontiers in Zoology 12, no. 1: 1–9. 28400852 10.1186/1742-9994-12-S1-S5 PMC5385817

[ece370569-bib-0027] Hepler, J. R. , W. Rodney Cooper , J. P. Cullum , et al. 2023. “Do Adult Magicicada (Hemiptera: Cicadidae) Feed? Historical Perspectives and Evidence From Molecular Gut Content Analysis.” Journal of Insect Science 23, no. 5: 13–14. 10.1093/jisesa/iead082 PMC10583540 37850668

[ece370569-bib-0028] Hertel, A. G. , A. G. Hertel , P. T. Niemelä , N. J. Dingemanse , T. Mueller , and T. Mueller . 2020. “A Guide for Studying Among‐Individual Behavioral Variation From Movement Data in the Wild.” Movement Ecology 8, no. 1: 1–18. 32612837 10.1186/s40462-020-00216-8 PMC7325061

[ece370569-bib-0029] Holm, S. , R. B. Davis , J. Javoiš , et al. 2016. “A Comparative Perspective on Longevity: The Effect of Body Size Dominates Over Ecology in Moths.” Journal of Evolutionary Biology 29, no. 12: 2422–2435. 27536807 10.1111/jeb.12966

[ece370569-bib-0030] Hopkins, J. , G. Baudry , U. Candolin , and A. Kaitala . 2015. “I'm Sexy and I Glow It: Female Ornamentation in a Nocturnal Capital Breeder.” Biology Letters 11, no. 10: 20150599. 26490414 10.1098/rsbl.2015.0599 PMC4650175

[ece370569-bib-0031] Hou, Z. , C. Luo , J. D. Roberts , and C. Wei . 2017. “Sexual Pair‐Formation in a Cicada Mediated by Acoustic Behaviour of Females and Positive Phonotaxis of Males.” Scientific Reports 7, no. 1: 1–10. 28743920 10.1038/s41598-017-06825-5 PMC5526892

[ece370569-bib-0032] Johnson, R. A. 2006. “Capital and Income Breeding and the Evolution of Colony Founding Strategies in Ants.” Insectes Sociaux 53, no. 3: 316–322.

[ece370569-bib-0033] Jönsson, K. I. , and K. I. Jonsson . 1997. “Capital and Income Breeding as Alternative Tactics of Resource Use in Reproduction.” Oikos 78, no. 1: 57.

[ece370569-bib-0034] Kemp, D. J. , and J. Alcock . 2003. “Lifetime Resource Utilization, Flight Physiology, and the Evolution of Contest Competition in Territorial Insects.” American Naturalist 162, no. 3: 290–301. 10.1086/376890 12970838

[ece370569-bib-0035] Koyama, T. , and C. K. Mirth . 2018. “Unravelling the Diversity of Mechanisms Through Which Nutrition Regulates Body Size in Insects.” Current Opinion in Insect Science 25: 1–8. 29602355 10.1016/j.cois.2017.11.002

[ece370569-bib-0036] Lee, Y. J. 2008. “Revised Synonymic List of Cicadidae (Insecta: Hemiptera) From the Korean Peninsula, With the Description of a New Species and Some Taxonomic Remarks.” Proceedings of Biological Society of Washington 121, no. 4: 445–467.

[ece370569-bib-0037] Lee, Y. J. , H. Y. Oh , and S. G. Park . 2004. “A New Habitat of *Cicadetta pellosoma* and *Cicadetta isshikii* (Hemiptera, Cicadidae) in Korea and Their Variations in Body Coloration.” Journal of Asia‐Pacific Entomology 7, no. 1: 127–131.

[ece370569-bib-0038] Luttbeg, B. 2017. “Re‐Examining the Causes and Meaning of the Risk Allocation Hypothesis.” American Naturalist 189, no. 6: 644–656. 10.1086/691470 28514637

[ece370569-bib-0039] McLachlan, A. , R. Ladle , and B. Crompton . 2003. “Predator–Prey Interactions on the Wing: Aerobatics and Body Size Among Dance Flies and Midges.” Animal Behaviour 66, no. 5: 911–915.

[ece370569-bib-0040] Molleman, F. , J. Granados‐Tello , C. A. Chapman , and T. Tammaru . 2022. “Fruit‐Feeding Butterflies Depend on Adult Food for Reproduction: Evidence From Longitudinal Body Mass and Abundance Data.” Functional Ecology 36, no. 8: 1961–1972.

[ece370569-bib-0041] Moschilla, J. A. , J. L. Tomkins , and L. W. Simmons . 2018. “State‐Dependent Changes in Risk‐Taking Behaviour as a Result of Age and Residual Reproductive Value.” Animal Behaviour 142: 95–100.

[ece370569-bib-0042] Niemelä, P. T. , and N. J. Dingemanse . 2018. “Meta‐Analysis Reveals Weak Associations Between Intrinsic State and Personality.” Proceedings of the Royal Society B: Biological Sciences 285, no. 1873: 20172823. 10.1098/rspb.2017.2823 PMC5832713 29491175

[ece370569-bib-0043] Niemelä, P. T. , E. Z. Lattenkamp , and N. J. Dingemanse . 2015. “Personality‐Related Survival and Sampling Bias in Wild Cricket Nymphs.” Behavioral Ecology 26, no. 3: 936–946.

[ece370569-bib-0044] Niemelä, P. T. , S. Tiso , and N. J. Dingemanse . 2021. “Density‐Dependent Individual Variation in Male Attractiveness in a Wild Field Cricket.” Behavioral Ecology 32, no. 4: 707–716.

[ece370569-bib-0045] Ory, N. C. , T. C. van Son , and M. Thiel . 2015. “Mating Rock Shrimp Hedge Their Bets: Old Males Take Greater Risk, but Only After Careful Assessment of the Investment Scenario.” Behavioral Ecology and Sociobiology 69, no. 12: 1975–1984.

[ece370569-bib-0046] Pöykkö, H. 2009. “Egg Maturation and Oviposition Strategy of a Capital Breeder, Cleorodes Lichenaria, Feeding on Lichens at the Larval Stage.” Ecological Entomology 34, no. 2: 254–261.

[ece370569-bib-0047] Rhainds, M. , S. R. Leather , and C. Sadof . 2008. “Polyphagy, Flightlessness, and Reproductive Output of Females: A Case Study With Bagworms (Lepidoptera: Psychidae).” Ecological Entomology 33, no. 5: 663–672.

[ece370569-bib-0048] Roitberg, B. D. , E. B. Mondor , and J. G. A. Tyerman . 2003. “Pouncing Spider, Flying Mosquito: Blood Acquisition Increases Predation Risk in Mosquitoes.” Behavioral Ecology 14, no. 5: 736–740.

[ece370569-bib-0049] Roth, A. M. , S. M. Kent , E. A. Hobson , G. Kritsky , and S. Nakagawa . 2022. “Personality‐Mediated Speed‐Accuracy Tradeoffs in Mating in a 17‐Year Periodical Cicada.” Behavioral Ecology 33, no. 6: 1141–1152.

[ece370569-bib-0050] Santostefano, F. , A. J. Wilson , Y. G. Araya‐Ajoy , and N. J. Dingemanse . 2016. “Interacting With the Enemy: Indirect Effects of Personality on Conspecific Aggression in Crickets.” Behavioral Ecology 27, no. 4: 1235–1246.

[ece370569-bib-0051] Satoh, S. , C. Iizuka , A. Kikuchi , N. Nakamura , and T. Fujii . 1992. “Proteins and Carbohydrates in Xylem Sap From Squash Root.” Plant & Cell Physiology 33, no. 7: 841–847.

[ece370569-bib-0052] Scriber, J. M. , and F. Slansky . 1981. “The Nutritional Ecology of Immature Insects.” Annual Review of Entomology 26, no. 1: 183–211.

[ece370569-bib-0053] Sih, A. , K. J. Mathot , M. Moirón , P. O. Montiglio , M. Wolf , and N. J. Dingemanse . 2015. “Animal Personality and State‐Behaviour Feedbacks: A Review and Guide for Empiricists.” Trends in Ecology & Evolution 30, no. 1: 50–60. 25498413 10.1016/j.tree.2014.11.004

[ece370569-bib-0054] Steward, V. B. , K. G. Smith , and F. M. Stephen . 1988. “Red‐Winged Blackbird Predation on Periodical Cicadas (Cicadidae: *Magicicada* spp.): Bird Behavior and Cicada Responses.” Oecologia 76, no. 3: 348–352. 28312013 10.1007/BF00377028

[ece370569-bib-0055] Tammaru, T. , and E. Haukioja . 1996. “Capital Breeders and Income Breeders Among Lepidoptera: Consequences to Population Dynamics.” Oikos 77, no. 3: 561.

[ece370569-bib-0056] White, J. , and C. E. Strehl . 1978. “Xylem Feeding by Periodical Cicada Nymphs on Tree Roots.” Ecological Entomology 3, no. 4: 323–327.

[ece370569-bib-0057] White, J. A. , and M. Lloyd . 1975. “Growth Rates of 17 and 13‐Year Periodical Cicadas.” American Midland Naturalist 94, no. 1: 127.

[ece370569-bib-0058] Williams, G. C. 1966. “Natural Selection, the Costs of Reproduction, and a Refinement of Lack's Principle.” The American Naturalist 100, no. 916: 687–690.

[ece370569-bib-0059] Wolf, M. , and F. J. Weissing . 2010. “An Explanatory Framework for Adaptive Personality Differences.” Philosophical Transactions of the Royal Society, B: Biological Sciences 365, no. 1560: 3959–3968. 10.1098/rstb.2010.0215 PMC2992748 21078648

