# Supplementary material for: Age‐Related Plasticity Integration in Male Cicadas Tettigetta isshikii: Are Adult Cicadas Capital Breeders?
Source: Ecol Evol. 2024 Nov 11;14(11):e70569. doi: 10.1002/ece3.70569 (PMC11554382; doi:10.1002/ece3.70569)
Supplement: Supplementary file 1 — Figures S1–S2. [file ECE3-14-e70569-s002.pdf]

Supplementary materials

Age-related plasticity integration in male cicadas *Tettigetta isshikii*: are adult cicadas capital breeders?

Jiman Heo [a] and Chang S. Han [a,b] *

[a] Department of Biology, Kyung Hee University, Seoul, 02447, Korea

[b] Korea Institute of Ornithology, Kyung Hee University, Seoul, 02447, Korea

* corresponding author: hcspol@gmail.com

Running title: Age-related plasticity integration

Figure S1. (A) The relationship between temperature and humidity measured during the observation. (B) Changes in humidity (blue dots) and temperature (red dots) over three daily observation periods (9 am, 12 pm, 5 pm) between 1 June and 13 June 2022.

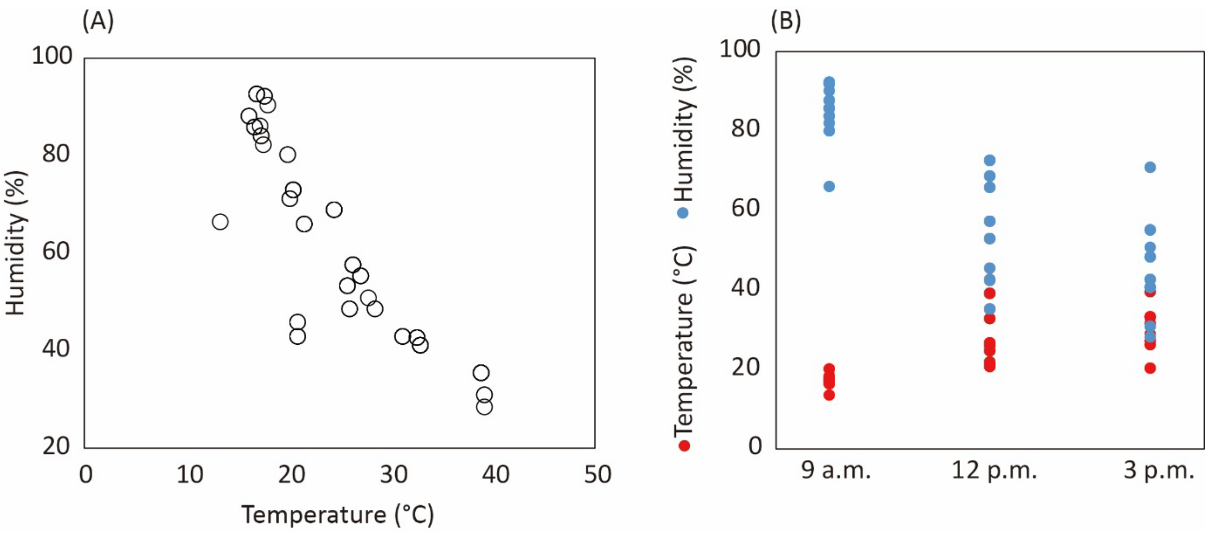

(a) plant use

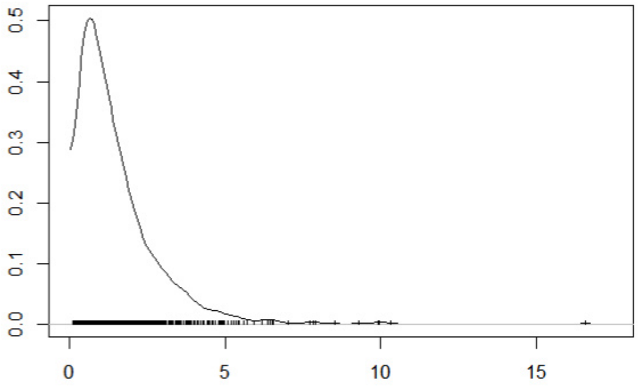

(b) calling activity

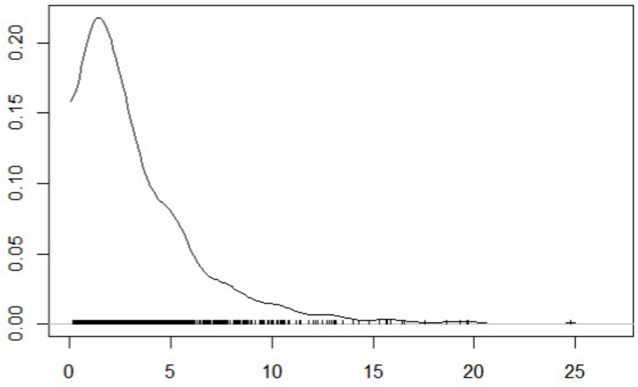

(c) body mass

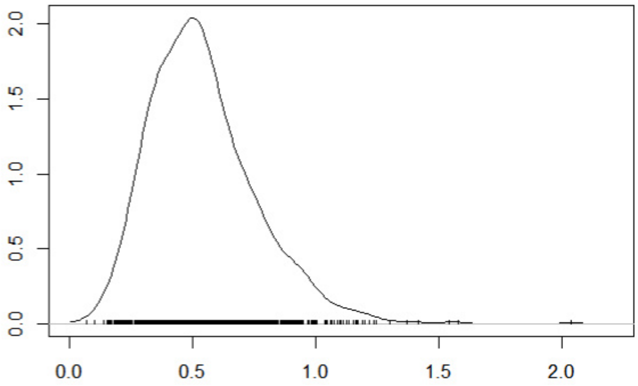

Figure S2. Plots represent the proportion of variance explained by male identity. Variance distributions were estimated through 1,000 bootstraps using GLMMs.
